# MicroRNA (miR) dysregulation during *Helicobacter pylori*-induced gastric inflammation and cancer development: critical importance of miR-155

**DOI:** 10.18632/oncotarget.27520

**Published:** 2020-03-10

**Authors:** Christian Prinz, David Weber

**Affiliations:** ^1^Lehrstuhl für Innere Medizin1, University of Witten gGmbH, Helios Universitätsklinikum, D-42283 Wuppertal, Germany

**Keywords:** Helicobacter pylori, gastric inflammation, gastric cancer, microRNA, miR-155

## Abstract

Dysregulation of noncoding microRNA molecules has been associated with immune cell activation in the context of *Helicobacter pylori* induced gastric inflammation as well as carcinogenesis, but also with downregulation of mismatch repair genes, and may interfere with immune checkpoint proteins that lead to the overexpression of antigens on gastric tumor cells. Numerous miR-molecules have been described as important tools and markers in gastric inflammation and cancer development —including miR-21, miR-143, miR-145, miR-201, and miR-335— all of which are downregulated in gastric tumors, and involved in cell cycle growth or tumor invasion. Among the many microRNAs involved in gastric inflammation, adenocarcinoma development and immune checkpoint regulation, miR-155 is notable in that its upregulation is considered a key marker of chronic gastric inflammation that predisposes a patient to gastric carcinogenesis. Among various other miRs, miR-155 is highly expressed in activated B and T cells and in monocytes/macrophages present in chronic gastric inflammation. Notably, miR-155 was shown to downregulate the expression of certain MMR genes, such as MLH1, MSH2, and MSH6. In tumor-infiltrating miR-155-deficient CD8^+^ T cells, antibodies against immune checkpoint proteins restored the expression of several derepressed miR-155 targets, suggesting that miR-155 may regulate overlapping pathways to promote antitumor immunity. It may thus be of high clinical impact that gastric pathologies mediated by miR-155 result from its overexpression. This suggests that it may be possible to therapeutically attenuate miR-155 levels for gastric cancer treatment and/or to prevent the progression of chronic gastric inflammation into cancer.

## INTRODUCTION: MICRORNA DYSREGULATION AND GASTRIC CANCER

Increasing evidence suggests that microRNA (miRNA) dysregulation has critical impacts on development, as well as inflammation and cancer development [1, 2]. Notably, it seems that human gastrointestinal cancer can be better classified using miRNA expression profiles than mRNA or protein expression profiles [3]. MicroRNAs are non-protein-coding RNAs of ~22 nucleotides, which induce translational repression and/or degradation of their mRNA targets. In complex with argonaute (AGO) proteins, miRNAs can use seed sequences near the 5′ end to base pair with a target mRNA, inducing deadenylation and decay or translational regulation [2]. Consequences of miRNA expression can include post-transcriptional silencing of targeted genes and thereby blocked translation of their target transcripts, and may also prevent apoptosis by binding to promoter units involved in cell cycle regulation. The functional roles of miRNAs are pleiotropic—for example, Let-7 miRNAs play key roles in development, stem cells, and cancer [4].

Lu et al. [3] recently reported that certain microRNAs can be used to profile tumors or tissues especially in gastrointestinal tumors, which have critical functions across various biological processes. Using a new bead-based flow cytometric miRNA expression profiling method, they performed a systematic expression analysis of 217 mammalian miRNAs from 334 samples, including multiple human cancers. Their results demonstrated the general downregulation of miRNAs in tumors compared with normal tissues. Furthermore, they successfully identified poorly differentiated tumors based on miRNA expression profiles, whereas classification of the same samples using messenger RNA profiles was highly inaccurate. These findings highlight the potential of miRNA profiling for cancer diagnosis.

Many miRNAs exhibit differential regulation in cancer—for example, miR-34a is involved in p53-mediated apoptosis in pancreatic cancer, and nine miRNAs are upregulated in primary breast cancer, including miR-21, miR-181b, and miR-155 [2, 4, 5]. Zhang et al. (2008) reported that miR-21 plays a pivotal role in gastric cancer pathogenesis and progression, and Yan et al. obtained similar data in breast cancer [6]. Our own group has highlighted the distinctive roles of miR-375 and miR-133a for discriminating rectal and colon cancer, respectively, and has demonstrated significant downregulation of these molecules in CRC [7]. Interestingly, growth factor analysis reveals that IGF-2 expression may be associated with these microRNA regulation patterns [8].

### MicroRNA dysregulation and gastric adenocarcinoma and related molecular targets

As outlined in Table 1, MicroRNA dysregulation seems to be of special importance in gastric adenocarcinoma (GC). The gastric carcinogenetic process is accompanied by numerous genetic and molecular changes, including oncogene activation, overexpression of growth factors, and inactivation of tumor suppressor and DNA repair genes. It has been postulated that miRNAs may play a role in this process, contributing to GC development as oncogenes or tumor suppressors, by directly or indirectly inhibiting the expression of target genes that may be involved in signaling pathways. For example, in GC, miRNA-221/222 reportedly counteracts PI3K signaling by modulating phosphatase and tensin homologue (PTEN) [9]. Additionally, miRNA-21 and miR-214 target PTEN, thereby increasing GC cell proliferation and invasion [10]. One of the most strongly downregulated miRNAs in gastric adenocarcinoma is miR-375, which targets a caspase-dependent apoptotic pathway, such that miRNA-375 expression reduces gastric cancer cell viability [11]. It has also been demonstrated that miRNA-143 regulates GC cell function in the PI3K/Akt pathway [12], and that miRNA-29s can influence cell cycle regulatory proteins (e. g., CDKs and cyclins) acting through a Ras/Raf/MEK/ERK pathway, and thereby facilitating cell cycle progression in GC [13]. Additionally, miRNA-107 expression is significantly decreased in GC, and its re-expression significantly decreases proliferation [14].

**Table 1:** Functional and biological roles of microRNAs in gastric cancer and related molecular targets, in the context of *H. pylori*, regulatory T cells, and cancer development

Name	Functional role	Biological role	Ref.
**miR-221** **miR-222**	miR-221 and miR-222 regulate radiosensitivity, cell growth, and invasion of GC cells, possibly via direct modulation of PTEN		[9]
**miR-21**	miR-21 inhibition may upregulate PTEN expression, indicating that PTEN may be a target gene for gastric cancer initiation and development	miR-21 expression is upregulated in GC and significantly associated with tumor differentiation, local invasion, and lymph node metastasis.	[10]
		miR-21 overexpression promotes GC cell growth, invasion, and migration *in vitro*, whereas miR-21 downregulation leads to an inhibitory effect	
**miR-375**	miR-375 expression inhibits expression of PDK1 (a direct target of miR-375) and 14-3-3zeta (a potent antiapoptotic gene), and suppresses Akt phosphorylation	miR-375 is downregulated in GC cells and reduces cell viability via the caspase-mediated apoptosis pathway through downregulation of PDK1 and 14-3-3zeta	[11]
	miR-375 may function as a tumor suppressor by targeting the JAK2 oncogene	miR-375 overexpression significantly inhibits gastric cancer cell proliferation, migration, and invasion	[16] [21]
**miR-143**	miR-143 and miR-145 act as anti-oncomers in GC	Expression levels of miR-143 and miR-145 are decreased in GC	[12]
**miR-145**	Possible candidate target mRNAs of	Overexpression leads to growth inhibition and higher sensitivity to 5-fluorouracil	
	miR-145 include insulin receptor substrate-1 and beta-actin		
**miR-181c**	miR-181c may be silenced through methylation and may play important roles in gastric carcinogenesis through its target genes, such as NOTCH4 and KRAS	miR-181c overexpression causes decreased growth of gastric cancer cell lines	[22]
**miR-29**	miR-29 targets Cdc42	miR-29 family molecules inhibit cell proliferation, migration, and invasion of gastric cancer cells by targeting Cdc42	[13]
**miR-107**	miR-107 may have a tumor suppressor function by directly targeting CDK6	Ectopic expression of miR-107 inhibits proliferation, induces G1 cell cycle arrest, and blocks invasion of gastric cancer cells	[14]
		miR-206 expression is significantly decreased in GC	[15]
**miR-206**	miR-206 is a potential tumor suppressor targeting cyclinD2 (CCND2)	miR-206 suppresses GC cell proliferation, and reduces cell growth and colony-forming ability via G0/G1 cell cycle arrest	
**miR-331**	miRNA-331-3p is a potential tumor suppressor in gastric cancer and directly targets E2F1	miR-331-3p overexpression blocks G1/S transition in GC cell lines, and suppresses colony-formation ability and cell growth *in vitro* by interfering with E2F1 activity	[17]

In GC, miRNA-206 appears to increase proliferation through modulation of the downstream target cyclin D2 [15]. Furthermore, miRNA-106b and miRNA-93 may be upregulated in GC and could be downstream targets of the oncogenic transcription factor E2F1, reducing the effectiveness of the tumor-suppressive function of transforming growth factor-β [16]. Interestingly, E2F1 seems to be a target gene of miRNA-331-3p and miRNA-106a, influencing cell cycle progression via increased G1/S-phase transition [17]. High miRNA-196a expression also appears to have particular clinical relevance, as it is associated with clinic-pathological parameters in GC, such as tumor size, poor pT stage, pN stage, and patients’ overall survival. Additionally, miRNA-375 may act as a tumor suppressor and regulate GC cell proliferation by targeting the JAK2 oncogene and janus kinases [16].

Recent research has also focused on microRNA dysregulation and the Wnt-catenin signaling pathway during the process of gastric inflammation and cancer development, and may be key to understanding the potential tumorigenic effects of microRNA deregulation in the process of gastric cancer. A recent study demonstrated that miR-194 inhibition suppressed the Wnt/β-catenin signaling pathway in gastric cancer [18]. In another study of gastric carcinoma, miR-23b-3p and miR-130a-5p appeared to affect cell growth, migration, and invasion by targeting CB1R via the Wnt/β-catenin signaling pathway [19]. Additionally, miR-381 and miR-489 have been found to decrease cell proliferation and invasion in gastric cancer by targeting CUL4B via the Wnt/β-catenin pathway [20].

### MicroRNA dysregulation in *Helicobacter pylori* induced gastric inflammation (see Table 2)

Let-7c expression was recently investigated in biopsy samples representing the whole spectrum of phenotypic changes involved in *H. pylori*-related gastric inflammation—including corpus gastritis, gastric atrophy, and intestinal metaplasia. Interestingly, Let-7c expression decreased from non-atrophic gastritis to atrophic-metaplastic gastritis, neoplasia, and invasive GC, and exhibited a significant increase following *H. pylori* eradication. Moreover, let-7c was downregulated in a mouse model following inoculation with *H. pylori*. Overall, these findings suggest that early stages of gastric disease are characterized by let-7c dysregulations [23].

**Table 2:** Differential dysregulation of microRNAs in early stages of gastric inflammation, gastric cancer tissues, and metastasis during *Helicobacter*-induced gastric adenocarcinoma

Inflammation	Carcinoma	Metastasis	Reference
miR-Let-7c	miR-Let-7c		[23]
	miR-Let-7b	miR-Let-7b	[24]
miR-106	miR-106		[25]
miR-375	miR-375		[25, 26]
	miR-490-3p	miR-490-3p	[27]
	miR-146a	miR-146a	[25, 26] [28, 29]
miR-155	miR-155	miR-155	[30]

A similar function has been described for Let-7b microRNA. Let-7b expression is downregulated in gastric adenocarcinoma, and shows correlations with *H. pylori* infection, tumor stage, and lymphatic metastasis. Ectopic expression of let-7b suppresses GC cell growth, migration, invasion, and tumorigenicity, whereas let-7b knockdown promotes these phenotypes. Interestingly, let-7b appears to directly target collagen triple helix repeat containing 1 (Cthrc1), which is negatively correlated with let-7b levels in GC. Overall, the available data suggest that let-7b may directly target Cthrc1 and function as a tumor suppressor gene in GC [24].

Additionally, downregulation of miR-375 and miR-106b has been detected in patients infected with *H. pylori*, and low expression of these microRNAs is correlated with inflammation scores and colonization density [25]. Prior studies show that miR-375 inhibits cell proliferation by targeting JAK 2 [16], and miR-375 downregulation has been observed in gastric cancer. It was recently reported that LPS from type 1 cagA+ strains of *H. pylori* increases MDM2 expression, yielding an autocrine feedback loop involving SP1/MDM2/p63/Dicer, and leading to inhibited miR-375 and miR-106b expression. JAK1 and STAT3 are downstream target genes of miR-106b, and are thus new targets within the carcinogenic process. Exposure to *H. pylori* LPS reportedly enhances tyrosine phosphorylation of JAK1, JAK2, and STAT3, potentially rendering cells susceptible to JAK1/JAK2 and STAT3 signal pathway activation via inhibition of miR-375 and miR-106b [26].

Of particular clinical relevance, SMARCD1 is markedly upregulated in the gastric tissues of patients with gastric inflammation and also gastric cancer, and high SMARCD1 expression is associated with shorter patient survival, independent of TNM staging [27]. Interestingly, miR-490-3p suppresses growth and metastasis in cell lines by targeting SMARCD1, a subunit of the chromatin remodeling complex. SMARCD1 knockdown significantly attenuates the pro-tumorigenic effects of miR-490-3p inhibitor. In this context, downregulation of miR-490-3p has been detected in gastric cancer tissues, along with miR-490-3p promoter hypermethylation, suggesting that the hypermethylation may lead to the downregulation of a potential tumor suppressor.

The deregulation of miR-146a also appears to be of special interest in the pathway of *H. pylori*-induced gastric inflammation as well as carcinogenesis. Gastric cancer exhibits clear miR-146a deregulation [28, 29], and recent evidence shows that miRNA-146a inhibits the inflammatory responses induced by interleukin-17A during *H. pylori* infection [29]. This inflammation-mediating role of miR-146a has been described and investigated in *H. pylori*-infected gastric tumors. Primary gastric tumors reportedly show miR-146a overexpression, which decreases in progressed tumors with higher stages and lymph node metastasis. However, these analyses reveal that miR-146a expression is independent of *H. pylori* infection. It has been suggested that miR-146a dysregulation promotes the progression of (later stages of) gastric tumorigenesis and thus promotes metastasis [28].

### MicroRNA dysregulation and mismatch repair deficiency in gastric cancer

Microsatellite instability (MSI) is found in a remarkably high proportion (15–30%) of gastric tumors. MSI is characterized by the accumulation of mutations at repetitive sequences (microsatellites) due to a defective DNA mismatch repair system (MMR) [31] or mutations in genes involved in the DNA damage response, such as ATR or CHK1 [32]. The MMR system comprises at least seven proteins, including MLH1, MLH3, MSH2, MSH3, MSH6, PMS1, and PMS2. These proteins associate with specific partners to form heterodimers that recognize base-pair mismatches and small nucleotide insertions/deletions that occur during DNA replication. In sporadic and familial gastric cancer exhibiting MSI, the leading mechanism of MMR deficiency is the epigenetic silencing of MLH1 by promoter methylation [33]. ATM mutations associated with impaired DNA repair function are also linked to an increased risk of gastric cancer [34], and are considered an independent prognosis factor in gastric cancer [35]. Thus, microRNAs may be a crucial regulatory mediator of the downregulation of DNA repair, and represent a potential tool for blocking the process of gastric malignant development, even after many years of chronic gastritis.

Accumulating evidence suggests that a close interaction between MSI and miRNA dysregulation plays a key role in the pathogenesis of GI cancer, as reviewed by Yamamoto et al. [36]. Overexpression of miR-155 reportedly downregulates the expression of MLH1, MSH2, and MSH6—potentially representing a new mechanism underlying MSI [37]. It is possible that miR-155 overexpression explains a subset of MSI-positive cancers without known MMR defects. Notably, MSI-positive GI cancers were found to have mutations in a novel class of target genes, including epigenetic modifier genes (e. g., HDAC2) as well as miRNA-processing machinery genes (e. g., TARBP2 and XPO5) [36].

Santos et al. recently found that *H. pylori* infection significantly downregulated the expression of almost all examined MMR genes, and investigated the possible role of microRNAs [38]. They demonstrated that *H. pylori*-infected mice exhibited altered expressions of miR-150-5p, miR-155-5p, and miR-3163 after several weeks of infection. Predictions of candidate miRs and their MMR targeting sites were obtained using TargetScan, and these predictions were confirmed by luciferase assays, indicating that miR-150-5p, miR-155-5p, and miR-3163 might target and modulate the MMR genes POLD3, MSH2, and MSH3, respectively [38]. Their findings strongly highlighted that certain miRs may impair the MMR-associated DNA repair pathway in gastric cancer. This suggests that targeting miR-155 overexpression (for example, via silencer RNAs) to allow DNA repair could be a promising method for controlling cancer growth, especially in pre-malignant lesions or during the early stages of gastric cancer.

### Role of microRNAs in immune checkpoint modulation

Immune checkpoint proteins (ICPs) are immune system regulators that can affect host immune responses to cancer-specific antigens, and thus contribute to the occurrence and progression of various cancers. Enormous clinical attention has been focussed on miRNAs that modulate immunity via immune checkpoint proteins, which seem to influence the outcome for gastric cancer and other cancer types, including certain skin cancers. In multiple types of cancer, miRNAs can directly or indirectly repress ICP expressions. Interestingly, miRNAs are also subject to regulation by ICPs themselves. Studies have revealed relationships between miRNAs and PD-1, PD-L1, CTLA-4, B7-1/2/H2, and other proteins. Further understanding of immune escape through microRNA dysregulation may allow miRNA-based guidance for personalized medicine, and predicting prognosis [39]. It has also been speculated that engagement of programmed death-ligand 1 (PD-L1) with its receptor programmed death 1 (PD-1) on T cells may play a major role in immune system suppression, enabling gastric cancer cells to escape host anti-tumor immunity. The development of whole genome sequencing technologies has increased the focus on microRNAs as an important layer of molecular regulation [40].

Interestingly, miR-155 plays a special role in the modulation of immune checkpoint regulation with regards to antitumor immune responses. miR-155 expression in T cells seems to limit tumor growth, and promote IFNγ production by T cells within the tumor microenvironment [41]. In miR-155 T-cell-conditional KO mice, antitumor immunity was restored by immune checkpoint-blocking (ICB) antibodies against programmed cell death protein 1/programmed death ligand 1 (PD-1/PD-L1) and cytotoxic T-lymphocyte-associated protein 4 (CTLA-4). Moreover, in tumor-infiltrating miR-155-deficient CD8^+^ T cells, antibodies against immune checkpoint proteins restored the expression of several derepressed miR-155 targets, suggesting that miR-155 and ICB regulate overlapping pathways to promote antitumor immunity. Overall, these findings highlight the potent capacity of miR-155 to promote antitumor immunity of T cells, and suggest that augmentation of miR-155 expression could potentially improve anticancer immunotherapies [41].

### MicroRNA dysregulation in *Helicobacter pylori*-induced inflammation: importance of regulatory T cells for cancer development

*H. pylori* infection is the principal cause of peptic ulcer disease, and the main risk factor for gastric cancer development [42–45]. Studies in humans demonstrate that regulatory T cells (Tregs) play a key role in the development of this specific immune response, which may lead to suppression of naturally induced immune responses in humans, thereby preventing eradication [46, 47]. Tregs are critical for the maintenance of self-tolerance, and react to environmental stimuli by modulating their cell surface and signaling molecules. Importantly, Treg dysfunction may lead to severe or even fatal autoimmune diseases [48–50]. In the later course of *H. pylori* infection, increased numbers of CD4^+^/CD25^+^ regulatory T cells (T_regs_) expressing Foxp3 are observed [51, 52]. Treg cells seem to have a similar function in the homeostasis of immune responses to other microbial infections, such as *Bordetella pertussis* and *Leishmania major,* preventing their eradication [48–50].

Studies in mice clearly show that *H. pylori* is primarily recognized through Toll-like receptors on dendritic cells. Myd88-deficient mice lacking a coupling of pattern recognition receptors do not react to *H. pylori* [47, 53], and various Toll-like receptor (TLR) subtypes are functionally important in this process [51, 52, 54]. Interestingly, microRNA dysregulation detected in gastric carcinogenesis has also been implicated in the process of Treg activation and depletion. Investigations of Treg depletion in mouse models have yielded the identification of numerous dysregulated miRNAs [55–57]. In patients with chronic inflammatory disorders, PCR and ROC curve analysis revealed three potential candidate miRNAs (miR-551b, miR-448, and miR-124), some of which were also detected in the chronically inflamed gastric tissues. These circulating miRNAs have been suggested as potential markers involved in autoimmune diseases induction [55–57], but do not appear to play an important role in gastric development.

A growing body of evidence indicates that regulatory T cells play a key role in the development of gastrointestinal malignancies, including gastric cancer [58]. Forkhead Box P3 (FoxP3) is considered a key transcription factor in Tregs, and is also expressed in several tumor cells [59]. However, its precise roles in gastric cancer remain unclear, an it is unknown what mechanisms underlie the interaction between gastric cancer cells and lymphocytes. Strong cytoplasmic staining of FoxP3 has been detected in gastric cancer cells [60], and low FoxP3 protein expression in these tumor cells predicts a good prognosis. On the other hand, high-density Treg presence predicts a poor prognosis in these cancer types. Accordingly, *H. pylori* infection induces high numbers of Tregs in the infected mucosa and in serum. Foxp3+ tumor-infiltrating lymphocytes (TILs) appear to be crucially important in the process of gastric cancer development, and thus may serve as a prognostic marker associated with chronic *H. pylori* infection [60]. Logistic regression models have revealed that a high FOXP3+ Treg density of the SLN is an independently significant predictor of metastasis [48, 61]. These results suggest the enormous clinical potential of Treg-targeting therapeutic agents in gastric cancer, and for regulating metastasis in gastric adenocarcinoma [48, 61].

Interestingly, the Tregs of different cancer types show different microRNA expression profiles [62], and potential miR target sites have been identified in the 3′-UTR of IL-10 (miR-27b-3p and miR-340-5p) and TGF-β (miR-330-3p). Additionally, miR-330-3p overexpression negatively regulates TGF-β expression [63]. Moreover, specific Tregs were recently assessed in EBV-associated gastric cancer, demonstrating that Tregs were significantly increased in EBV-positive gastric cancer compared to EBV-negative gastric cancer. It was postulated that this Treg accumulation may be promoted by decreased emigration due to CCR7 downregulation on the Treg surface, and the lower apoptosis rate of Tregs at tumor sites.

### Prognostic value of microRNA in gastric tissue and cancer, and as a serum biomarker

Only few data exist which indicate that microRNA dysregulation can predict a poor prognosis in patients suffering from advanced gastric cancer. In serum specimens from patients with gastric cancer, dysregulation of miR-195-5p and miR-218-5p seems to indicate a poor prognosis, acting through the BIRC-r pathway [64]. Additionally, miR-1297 downregulation reportedly predicts poor prognosis in gastric cancer, via targeting of CREB1 [65]. Dysregulation of the miR-532/NCF2-NF*k*B feedback loop promotes gastric cancer angiogenesis and metastasis [66], and dysregulation of the miR-126/Crk protein axis predicts poor prognosis in gastric cancer [67]. Altered miR-128/SNAIL signaling in gastric cancer reportedly regulates growth, invasion, metastasis, and epithelial-to-mesenchymal transition [68], and miR-99 dysregulation induces gastric cancer cell migration and invasion [69]. Song et al. recently demonstrated that gastric cancer chemoresistance is related to overexpression of excision repair cross-complementing 1 (ERCC1), caused by microRNA-122 dysregulation [70]. However, although numerous microRNAs may predict a poor prognosis in cancer, none of these markers has been specifically associated with the pathogenesis of inflammation, or has been detected in early stages of gastric cancer. Such a prognostic miRNA is sought, as it might allow the prediction of cancer development in patients chronically infected with *H. pylori*, a well-known type I carcinogen. miR-155 may be a special candidate to fulfill these criteria.

### Summary: critical role of miR-155

Among the many microRNAs involved in gastric adenocarcinoma development, progression, and immune checkpoint regulation, miR-155 is notable in that its upregulation is considered a key marker of chronic gastric inflammation that predisposes a patient to gastric carcinogenesis. Additionally, miR-155 is highly expressed in activated B and T cells and in monocytes/macrophages, and recent data indicate that miR-155-5p plays critical roles in various physiological processes, including immunity, inflammation, cancer development, and cardiovascular disease. It may be of high clinical impact that pathologies mediated by miR-155 result from miR-155 overexpression. This suggests that it may be possible to therapeutically attenuate miR-155-5p levels for gastric cancer treatment and/or to prevent the progression of chronic gastric inflammation into cancer [71].

The miR-155 knockout mouse model is useful for studying the roles of T-cell activation and differentiation. Detailed analysis has revealed that miR-155 plays a T-cell-intrinsic role in TH17-cell differentiation, as well as an indirect role by regulating DCs’ production of TH17-cell-polarizing cytokines [72]. Impaired TH17-cell responses of miR-155-deficient T cells have been described in a mouse model of *H. pylori* infection, and in a mouse model of TH17-driven chronic colitis [73]. Thus, it appears that miR-155 controls T-cell-mediated tissue inflammation through the regulation of both TH1-cell and TH17-cell responses. In contrast, upregulation of miR-301a enhances TH17-cell differentiation, possibly by targeting PIAS3, which inhibits STAT3 signaling and TH17-cell differentiation [74]. Overall, the data suggest that miR-155 may play important roles in the activation of regulatory T cells and the suppression of immune responses.

Induction of miR-155 expression has been observed upon *H. pylori* infection of gastric epithelial cells [30], and miR-155 expression is present in gastric adenocarcinoma [30]. The regulation of miR-155 is dependent on the activator protein 1 (AP-1) pathway in B cells [75] and in primary murine macrophages [76]. Importantly, increased Foxp3 reportedly controls miR-155 expression in T cells [30], and bacterial LPS exposure induces miR-155, miR-132, and miR-146a expression in immune cells [77]. Numerous studies in murine macrophages and C57Bl6 mice have demonstrated the induction of miR-155 expression in primary macrophages by a range of toll-like receptor ligands [78]. Given the specific importance of miR-155 in the regulation of gastric cancer development, this microRNA may be a critical determinant of gastric cancer development prior to tumor growth and metastasis. Recently published findings suggest that microRNAs, such as miR-155, become activated in gastric immune cells following *H. pylori* infection. Activation of Foxp3+ Tregs and noncoding miR-155, which are associated with activation of the signal transduction factor Stat3, may serve as prognostic factors in patients with chronic inflammation that predisposes them to development and progression of gastric cancer and metastasis.

**Figure 1:**
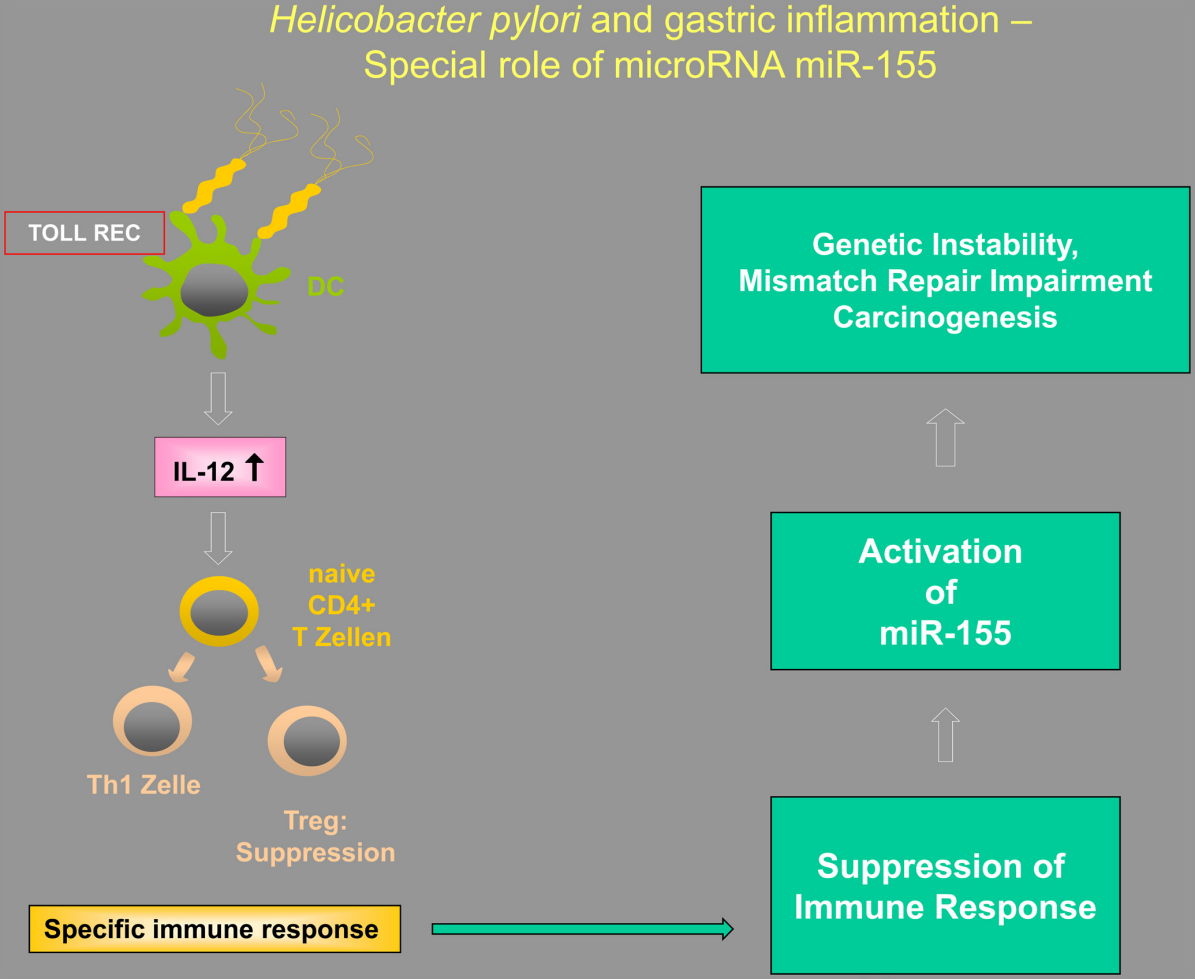
Illustration of the putative interactions between Tregs, Toll-like receptors (TLRs), and microRNAs in *Helicobacter*-induced inflammation, highlighting the special importance of miR-155.

Figure 1 presents an overview of the sequential events involved in this immunological response towards *H. pylori*. The pathogenic persistence is presumably facilitated through the induction of tolerance. *H. pylori* activates regulatory T cells in the stomach through the activation of TLR on dendritic cells, and these regulatory T cells might play important roles in cancer development and metastasis. The currently available data highlight an inverse relation between Tregs and cancer. High FoxP3 expression in tumor cells predicts worse survival in gastric cancer, possibility related to interactions between tumor cells and lymphocytes in the microenvironment. Recent immunological observations suggest that in the context of *H. pylori* infection, dysregulation of certain miRNAs, such as miR-155, is of critical importance in malignant pathways, and leads to deregulation of genes important for controlling genomic stability. Clinical strategies aiming to prevent miR-155 overexpression (i. e., via silencer RNAs) may thus represent a promising method of controlling cancer growth (e. g., by allowing DNA repair), especially in pre-malignant lesions or during the early stages of gastric cancer.
